# LC‐ESI‐QTOF‐MS/MS Characterization of Phenolic Compounds in the Stem, Roots, and Leaves of *Syzygium cumini* and Their Antioxidant Potential

**DOI:** 10.1002/fsn3.70112

**Published:** 2025-04-01

**Authors:** Ali Imran, Shujun Ye, Jiaying Amanda Li, Rahaf Ajaj, Abdur Rauf, Zubair Ahmad, Hassan A. Hemeg, Yahya Saleh Mohamed Al‐Awthan, Omar S. Bahattab, Mohammed Mansour Quradha, Hafiz Suleria

**Affiliations:** ^1^ School of Agriculture, Food and Ecosystem Sciences, Faculty of Science The University of Melbourne Parkville Victoria Australia; ^2^ Department of Food Science, Faculty of Life Science Government College University Faisalabad Pakistan; ^3^ Department of Environmental and Public Health, College of Health Sciences Abu Dhabi University Abu Dhabi UAE; ^4^ Department of Chemistry University of Swabi Swabi Pakistan; ^5^ Department of Clinical Laboratory Technology, College of Applied Medical Sciences Taibah University Madinah Saudi Arabia; ^6^ Department of Biology, Faculty of Science University of Tabuk Tabuk Saudi Arabia; ^7^ Biodiversity Genomics Unit, Faculty of Science University of Tabuk Tabuk Saudi Arabia; ^8^ College of Education Seiyun University Seiyun Yemen; ^9^ Pharmacy Department, Medical Sciences Aljanad University for Science and Technology Taiz Yemen

**Keywords:** antioxidants, LC‐ESI‐QTOF‐MS/MS, *Syzygium cumini*, therapeutic potential

## Abstract

*Syzygium cumini* , commonly known as Jamun or Indian Blackberry, is a fruit‐bearing tree native to the Indian subcontinent, revered for its medicinal uses, and characterized by a rich phytochemical profile abundant in polyphenols. This study aimed to characterize various fractions, including the roots, stem, and leaves of *S. cumini* (SC). Purposefully, conventional extraction was carried out by using ethanol (70%) with formic acid (1%) to extract the phytochemicals. The resultant extracts were subjected to phenolic contents (TPC, TFC, TTC) and antioxidant activity estimation (DPPH, FRAP, ABTS RPA, OH, FICA, and TAC). Further, LC‐ESI‐QTOF‐MS/MS identification of phenolics was also performed. The outcomes showed that *S. cumini* leaf exhibited the highest phenolic content and antioxidant activity among different fractions compared to stem and root. The recorded TPC, TFC, and TTC in the leaf were 52.17 ± 1.60 mg GAE/g, 2.76 ± 0.054 mg QE/g, and 17.22 ± 0.43 mg ce/g, respectively. Correlation analysis revealed a strong positive correlation (0.50 < *r* < 0.80, *p* < 0.01) between TPC, TFC, and TTC. The LC‐ESI‐QTOF‐MS/MS screening showed the presence of 12 compounds in the stem, leaf, and root of *S. cumini* , showing the diversity of phenolics between different parts. The majority of the compounds belonged to flavonoids (4), phenolic acids (3), other polyphenols (3) and lignans (2). Among the notable compounds, naringin 4'‐O‐glucoside, 3,4‐*O*‐dimethylgallic acid, scutellarein, and demethyloleuropein were identified, highlighting the therapeutic potential of different fractions of *S. cumini.* In conclusion, the results indicated that SC fractions contained a considerable amount of phenolics, thus showcasing higher antioxidant activity. Moreover, the concentration of different phenolics varied among fractions, as confirmed through a Venn diagram.

## Graphical abstract

Here we investigated the phytochemical profile and antioxidant potential of various fractions (roots, stem, and leaves) of *Syzygium cumini* . The leaf fraction showed the highest phenolic content and antioxidant activity. LC‐ESI‐QTOF‐MS/MS analysis identified 12 phenolic compounds, highlighting the therapeutic potential of *S. cumini* fractions.

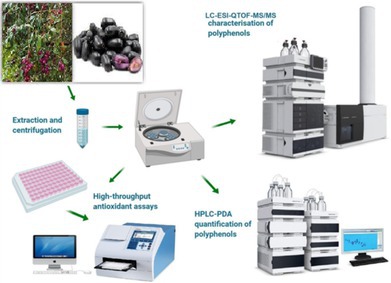

## 1 Introduction

The genus *Syzygium*, belonging to the family Myrtaceae, encompasses a diverse group of flowering plants with considerable ecological and economic significance. Among the species within this genus is *Syzygium cumini* (L.) Skeels, commonly known as Jamun or Java plum, holds particular importance due to its extensive traditional uses in various medicinal and culinary applications (Ahmed et al. 2019). The different parts of the *S. cumini* plant, including stem, root, and leaf, have long been recognized for their therapeutic potential, prompting scientific interest in elucidating the bioactive constituents responsible for these beneficial effects (Minabi‐Nezhad et al. 2023).

The therapeutic attributes of *S. cumini* have been attributed to its richness of phenolic compounds, which are secondary metabolites known for their diverse pharmacological activities, including antioxidant properties (Minnady et al. 2022). According to the latest research, the metabolic pathway through the phenolic compounds produced in *Syzygium cumini* is the shikimate pathway (Chakraborty et al. 2023). Antioxidants serve a crucial function in neutralizing reactive oxygen species (ROS) and reducing oxidative stress, a process implicated in various chronic diseases. Given the significance of phenolic compounds in mediating antioxidant effects, a comprehensive investigation into the phenolic profile of different plant parts of *S. cumini* becomes imperative (Namraaziz et al. 2019).

*S. cumini* has garnered attention for its diverse therapeutic applications in traditional medicine. Widely recognized for its anti‐diabetic properties, *S. cumini* has been traditionally employed to manage diabetes mellitus. The occurrence of bioactive phytochemicals like anthocyanins, ellagic acid, and quercetin in various parts of the plant contributes to its potential hypoglycemic effects (Gupta and Sharma 2017). Moreover, the plant's pharmacological attributes extend to anti‐inflammatory, antioxidant, and antimicrobial activities, showcasing its versatility in addressing multifaceted health challenges. Given the escalating global burden of chronic diseases, exploring the holistic therapeutic potential of *S. cumini* becomes increasingly pertinent, potentially paving the way for the development of novel, nature‐inspired remedies for a spectrum of health conditions (Sukmaningsih et al. 2019).

The therapeutic importance of *S. cumini* extends beyond its traditional applications, encompassing a myriad of health benefits associated with its distinct plant parts. The stem of *S. cumini* has been historically utilized in folk medicine for its anti‐diabetic properties. Research indicates that the stem extract exhibits hypoglycemic effects. These findings underscore the potential of *S. cumini* stem as a natural remedy for managing diabetes, a chronic metabolic disorder with increasing global prevalence (Kumar et al. 2022).

The root of *S. cumini* also contributes significantly to its therapeutic profile. Studies suggest that the root extracts possess anti‐inflammatory properties, attributed to the presence of triterpenoids and flavonoids (Shrikant Baslingappa et al. 2012). Inflammation is a hallmark of various chronic diseases, and the anti‐inflammatory potential of *S. cumini* root highlights its possible application in mitigating inflammatory conditions (Singh et al. 2019).

Liquid Chromatography‐Electrospray Ionization‐Quadrupole Time‐of‐Flight Mass Spectrometry (LC‐ESI‐QTOF‐MS/MS) has emerged as a useful technique for the characterization of complex mixtures of phytochemicals. Its high sensitivity and ability to provide precise mass measurements make it an excellent and ideal tool for identifying and quantifying phenolic compounds in plant extracts. Using LC‐ESI‐QTOF‐MS/MS, researchers can obtain valuable insights into the chemical composition of *S. cumini* and its antioxidant potential (Ahmad et al. 2024).

Several studies have highlighted the free radical scavenging, anti‐inflammatory, and anti‐diabetic properties of *S. cumini* extracts (Kumar et al. 2022). However, a detailed characterization of the phenolic constituents in different plant parts using LC‐ESI‐QTOF‐MS/MS is currently lacking. Thus, this study uses freeze‐dried samples to extract the phenolic compound by conventional extraction, analyzing the antioxidant ability while focusing on a systematic investigation of the phenolic composition of the stem, roots, and leaf of *S. cumini* by LC‐ESI‐QTOF‐MS/MS. We intend to identify and quantify individual phenolic compounds present in each plant part through a comprehensive analysis. Furthermore, the antioxidant potential of these compounds would be assessed to perform a holistic understanding of the medicinal properties associated with different plant components.

## 2 Materials and Methods

### 2.1 Sample Preparation

The stem, root, and leaf of *S. cumini* were collected in July from Old Campus University of Swabi, Khyber Pakhtunkhwa, Pakistan and were identified by Dr. Muhammad Ilyas, a famous plant taxonomist. The voucher specimen, labeled UOS/Bot‐103, was kept in the department's herbarium. The plant material was cut into 0.5 × 1 cm pieces and frozen overnight at −20°C. These samples were lyophilized at −45°C/50 MPa. An electric grinder was used to grind the freeze‐dried sample into a fine powder stored at −20°C.

### 2.2 Extraction of Phenolic Compounds

2.0 ± 0.5 g of each part (stem, root, and leaf) of *S. cumini* powder was blended with 20 mL of 70% ethanol using the conventional extraction method. Homogenization was performed to ensure the sample and solvent were mixed well. The first step of extraction was for 16 h at 120 rpm under 10°C in an incubator shaker. The supernatant, stored at −20°C, was collected after centrifugation at 5000 rpm for 15 min for the following analysis.

### 2.3 Phenolic Compounds and Antioxidant Assays

A total of ten assays were conducted in this section, including three phenolic content estimations: TPC (total phenolic content), TFC (flavonoids content in total), and TTC (tannins total concentration). Additionally, seven different antioxidant assays were performed: 2,2‐Diphenyl‐1‐picrylhydrazyl (DPPH), ferric reducing antioxidant ability assay (FRAP), 2,2′‐azino‐bis(3‐ethylbenzothiazoline‐6‐sulfonic acid) scavenge ability (ABTS), reducing power assay (RPA), hydroxyl (•OH‐) radical scavenging assay (·OH‐RSA), ferrous ion chelating ability (FICA), and total antioxidant assay(TAC). The methodology was adapted from Tang et al. (Tang et al. 2019).

#### 2.3.1 TPC

The mechanism of TPC was the colorimetric reaction between phenolic compounds and Folin–Ciocâlteu reagent (FCR) under an alkaline environment. The modification method was used by Severo et al. (2011) with minor modifications. The entire experimental procedure was conducted on a 96‐well plate. Mixing 25 μL of the extract (100 μg/mL) with 25 μL of diluted FCR (1:3 in water) and 200 μL of water was performed. This was followed by a 5 min incubation in darkness at 25°C. After adding 10% Na_2_CO_3_, a 60 min incubation of the mixture followed. Using gallic acid as standard (0–200 mg/mL) to calculate the standard equivalent (GAE) of the sample under 743 nm by a microplate reader.

#### 2.3.2 TFC

The TFC was using a method marginally modified from Danying et al. (2019). The entirety of the experimental process took place within a 96‐well plate. A combination of 80 μL of *S. cumini* extraction, 80 μL of 2% AlCl_3_, and 120 μL of 50 mg/mL CH_3_COONa solution was incubated in darkness for 2.5 h at room temperature (25°C). A wavelength of 440 nm was used to measure TFC absorbance, with a quercetin calibration curve ranging from 0 to 50 μg/mL.

#### 2.3.3 Determination TTC

The TTC assay was conducted following the standard procedure described by Zou et al. (Zou et al. 2014). Briefly, the mixture of 25 μL *S. cumini* extract, 150 μL vanillin (1:25 ratio in methanol), and 25 μL sulfuric acid (8:25 ratio in methanol) was incubated for 15 min at room temperature in the dark. The spectrum is 500 nm, with 0–1 mg/mL catechin as the standard to calculate the standard equivalent (CAE).

#### 2.3.4 DPPH Assay

The method generated by Hasan et al. (2009) with some modifications was the reference method of the DPPH assay. It was conducted in the 96‐well plate, which involved mixing 40 μL of extract (100 μg/mL) with 260 μL of a 0.1 mM DPPH solution. The mixture was incubated in the dark at 25°C for 40 min, after which the absorbance was calculated at 517 nm. Milligram of Trolox equivalent per gram of sample (mg TE/g) was the outcome of preparing a Trolox standard curve with a range of 0 to 0.2 mg/mL.

#### 2.3.5 FRAP Assay

The FRAP assay was conducted following a previously established standard method of Benzie and Strain (1996). The Trolox range from 0 to 0.2 mg/mL was used as a standard. The preparation of FRAP dye is the combination of 0.3 M sodium acetate, 0.01 M TPTZ, and 0.02 M FeCl_3_ with a 10:1:1 volume ratio. The mixture of 20 μL extracts and 280 μL FRAP dye was read for absorbance at 593 nm after 10 min of incubation at 37°C. The results were expressed as Trolox equivalents per gram of dry weight.

#### 2.3.6 ABTS Assay

This assay was conducted in a 96‐well plate as described by Tang, et al. (Tang et al. 2019) with some modifications. In brief, 1.25 mL 7 mM ABTS and 22 μL 0.14 M potassium persulfate were used to prepare the ABTS stock. The stock should be kept in a dark environment for at least 24 h at room temperature. The ABTS dye is prepared by diluting the stock with a 1:90 ratio of ethanol. The mixture of 10 μL extract and 290 μL dye was placed in a 96‐well plate for 6 min of incubation, and the absorbance was measured under 734 nm with ascorbic acid as standard (0–150 μg/mL).

#### 2.3.7 RPA

The reducing power of the extracts was measured by a well‐known procedure described by Ferreira et al. 2007; (Ferreira et al. 2007) with some modifications. 0.2 M Na_2_HPO_4▪_ 7H_2_O and 0.2 M NaH_2_PO_4▪_ H_2_O prepare sodium phosphate buffer with a 3:5 volume ratio. The whole procedure occurred in a 96‐well plate at room temperature, 10 μL extract, 25 μL sodium phosphate buffer, and 25 μL of 1% (w/v) K_3_[Fe(CN)_6_] were incubated for 20 min, then adding 25 μL 10% trichloroacetic acid, 85 μL water, and 8.5 μL 0.1% FeCl_3_ to incubate for another 15 min. The absorbance was read under 750 nm with a microplate reader. 0–0.5 mg/mL Trolox was used as a standard.

#### 2.3.8 •Oh‐RSA

The Smirnoff (Smirnoff and Cumbes 1989) method with modifications has been used to determine the hydroxyl radical scavenging ability of the extracts. For this assay, the combination of extracts, 6 mM FeSO_4_·7H_2_O, and 6 mM H_2_O_2_, with a 1:1:1 v/v ratio, was incubated at 25°C for 10 min. Following this, the same volume of 6 mM 3‐hydroxybenzoic acid was added, recording the absorbance at 510 nm. The scavenging ability was conducted by using an ascorbic acid standard curve that ranged from 0 to 0.3 mg/mL, with results expressed as mg AAE/g.

#### 2.3.9 FICA Assay

Based on the method described by Dinis (Dinis et al. 1994) the chelating activity of ferrous ions was determined. A combination of extract, water, ferrous chloride, and ferrozine was incubated at 25°C for 10 min. 562 nm is the wavelength to measure the absorbance. An Ethylenediaminetetraacetic acid (EDTA) standard curve, ranging from 0 to 50 μg/mL, was used to calculate the chelating activity, with results expressed as milligrams of EDTA per gram (mg EDTA/g).

#### 2.3.10 Tac

Referring to Jan et al. (2013), the total antioxidant potential of the samples was evaluated using the phosphomolybdate method. A TAC dye solution was the combination of 0.6 M sulfuric acid, 28 mM sodium phosphate, and 4 mM ammonium molybdate. 40 μL of the sample extract was added to 260 μL of the dye solution in a 96‐well plate, which was then placed in an oven at 95°C for 90 min and cooled to room temperature. Following the cooling period, the absorbance was assessed at 765 nm. The Trolox calibration curve ranged from 0 to 0.2 mg/mL, and the results were taken as mg TE/g.

### 2.4 LC‐ESI‐QTOF‐MS/MS

The LC‐ESI‐QTOF‐MS/MS analysis was performed for the identification and characterization of the phenolic compound according to the study by Suleria et al. (Suleria et al. 2020). The instrument is from Agilent Technologies. The separation of the compounds was achieved by a Synergi Hydro‐RP 80 Å, reverse phase column (250 mm × 4.6 mm, 4 μm particle size) with a protected C18 ODS guard column (4.0 × 2.0 mm), which was purchased from Phenomenex.

Filtration of extracted phenolic compounds by 0.45 μm syringe filter, then transferred into vials. Mobile phases were prepared as follows: A was a mixture of water and acetic acid at a 99.5:0.5 (v/v) ratio, while B was the combination of acetonitrile, water, and acetic acid with a 50:49.5:0.5 (v/v/v) ratio. Both need to undergo degassing at 21°C for 15 min. The injection volume is 5 μL with a 0.8 mL/min flow rate. ESI was employed in both positive and negative modes, and the mass spectra were recorded in the *m/z* range of 50–1300. The ingredients information was as follows: 0.0–30 min, 90%–65% buffer A; from 30 to 35 min, 65%–60% buffer A; from 35 to 40 min, 60%–45% buffer B; from 40 to 50 min, 45%–24% buffer A; from 50 to 55 min, 25%–5% buffer A; from 55 to 57 min, 50% buffer A; from 57 to 60 min, 0%–90% buffer A.

### 2.5 Statistical Analysis

All results are presented as mean ± standard deviation (*n* = 3), with all analyses conducted in triplicates. One‐way analysis of variance (ANOVA) and Tukey's honestly significant differences (HSD) multiple rank test at *p* ≤ 0.05, which was carried out by Minitab for Windows version 19.0 (Minitab LLC), were performed the mean differences between different samples. Pearson's correlation coefficient (*p* ≤ 0.05) and multivariate statistical analysis were used to evaluate correlations between polyphenol content and antioxidant activities; XLSTAT (2019.1.3, Addinsoft Inc.) was used. Agilent Mass Hunter Data Acquisition Software (Version B.03.01) was used for data acquisition and analysis for LC‐ESI‐QTOF‐MS/MS.

## 3 Results and Discussion

### 3.1 Phenolic Compound Estimation

In the present study, the phenolic composition of various parts of *S. cumini* , namely stem, roots, and leaves, was determined, with all the results summarized in Table 1. The importance of natural polyphenols in functional foods has been widely acknowledged for their antimicrobial, anti‐inflammatory, and antiviral effects. Similarly, *S. cumini* , known for its traditional uses, reveals a rich profile of phenolic compounds with potential health benefits.

**TABLE 1:** Polyphenol content and antioxidant ability detected in conventional extracts of stem, root, and leaf of *S. cumini* .

Sample	TPC (mg GAE/g)	TFC (mg QE/g)	TTC (mg ce/g)	DPPH (mg TE/g)	ABTS (mg AAE/g)	FRAP (mg TE/g)	RPA (mg TE/g)	·OH‐RSA (mg AAE/g)	FICA (mg EDTA/g)	TAC (mg TE/g)
*S. cumini* S.C (Stem)	33.86 ± 2.57^B^	1.12 ± 5.89^B^	10.21 ± 0.17^B^	66.14 ± 1.31^A^	22.88 ± 0.911^AB^	36.60 ± 1.58^B^	57.87 ± 3.03^B^	87.57 ± 7.51^B^	0.042 ± 0.076^B^	4.41 ± 0.001^A^
*S. cumini* S.C (Roots)	25.11 ± 1.40^C^	0.77 ± 1.45^C^	10.82 ± 0.54^B^	32.51 ± 0.64^B^	20.57 ± 0.199^B^	33.22 ± 0.927^B^	28.45 ± 1.88^C^	348.52 ± 18.55^A^	0.066 ± 0.088^A^	1.20 ± 0.02^B^
*S. cumini* S.C (Leaves)	52.17 ± 1.60^A^	2.76 ± 0.054^A^	17.22 ± 0.43^A^	67.87 ± 2.27^A^	24.59 ± 0.899^A^	59.61 ± 0.941^A^	71.23 ± 6.92^A^	—	0.067 ± 0.096^A^	5.99 ± 0.001^A^

*Note:* All values are expressed as mg/g mean ± standard deviation (*n* = 3); ^A,B^indicate the means in a row with significant differences (*p* < 0.05) using a one‐way analysis of variance (ANOVA) and Tukey's test.

Phenolic compounds, flavonoid content, and tannins are integral components of plant‐derived substances known for their significant therapeutic value. Phenolics, encompassing a diverse range of compounds like flavonoids, phenolic acids, and tannins, exhibit potent antioxidant properties. Their capacity to scavenge free radicals promotes overall health while helping prevent oxidative stress‐related diseases (Boateng and Clark 2024). Total flavonoid content, representing a subgroup of phenolic compounds, is recognized for its anti‐inflammatory, anti‐allergic, and anti‐cancer properties. Flavonoids play a crucial role in modulating various cellular pathways, positively influencing human health. On the other hand, total tannin content, known for its astringent properties, has been associated with anti‐bacterial, anti‐viral, and anti‐inflammatory effects. The therapeutic synergy of phenolics, flavonoids, and tannins underscores their potential as natural remedies with a range of health advantages, generating continued interest in them in the fields of medicine and nutrition (Boateng and Clark 2024; Lu et al. 2024).

The total phenolic content (TPC) assay revealed that the leaves of *S. cumini* exhibited the highest phenolic content (52.17 ± 1.60 mg GAE/g), followed by the stem and roots. This trend aligns with numerous studies emphasizing the antioxidant potential of plant leaves. The TFC and TTC assays also demonstrated variations among the plant parts, with the leaves consistently displaying higher values. The investigation into TFC revealed that the leaves of *S. cumini* displayed the highest content (2.76 ± 0.054 mg QE/g), emphasizing their potential as a rich source of flavonoids. On the other hand, the roots exhibited the lowest TFC value (0.77 ± 1.45 mg QE/g). This pattern is in harmony with existing literature, which often associates higher TFC with plant parts that contribute significantly to antioxidant and anti‐inflammatory properties. Similarly, the analysis of TTC demonstrated a notable variation among the plant parts. The leaves recorded the highest TTC (17.22 ± 0.43 mg ce/g), featuring their potential as a source of condensed tannins. In contrast, the stem exhibited the lowest TTC (10.21 ± 0.17 mg ce/g). This finding suggests that different parts of *S. cumini* possess distinct phenolic profiles, contributing to the plant's overall antioxidant potential.

The outcomes of this work are in line with the earlier findings. For instance, Ahmed et al. (Ahmed et al. 2019) studied the phenolic and therapeutic characterization of *S. cumini* through various in vitro assays like TPC, TFC, DPPH, etc. They observed that the methanolic content exhibited promising TPC activity of 369.75 ± 17.9 mg GAE/g alongside flavonoid contents of 75.8 ± 5.3 mg RE/g. Similarly, Saraswathy et al. (Benherlal and Arumughan 2007) exhibited the complete antioxidant profiling of *S. cumini* such as pulp, kernel, and seed coat, and narrated that all parts exhibited good TPC and TFC contents; however, the order of effectiveness was pulp > kernel > seed, with values of 340.0 ± 1.7, 370.0 ± 7.8, and 270.0 ± 3.4, respectively. The examination of phytochemical constituents in *S. cumini* unveiled the presence of different phytochemicals like flavonoids and phlobatannins, causing higher TPC activities. Phenols hold significance in plants owing to their ability to scavenge radicals, with their scavenging activity attributed to the hydroxyl groups within phenolic compounds. The phenolic content of plants is directly associated with their antioxidative activity (Ugbabe et al. 2010). Moreover, Patel & Rao (Patel and Rao 2012) observed that the *S. cumini* leaves were found to harbor additional bioactive compounds such as gallic acid, citric acid, carotenoids, malic acid, alkaloids, and polyphenols, recognized for their efficacy in medicinal systems. Determination of Antioxidant activity.

The DPPH assay determined the antioxidant potential of different parts of *S. cumini* . Table 1 shows that the DPPH values varied among the stem, roots, and leaves. The results revealed that the leaves of *S. cumini* exhibited the highest DPPH value (67.87 ± 2.27 mg TE/g), indicating a significant free radical scavenging capacity. In contrast, the stem and roots displayed lower DPPH values (66.14 ± 1.31 mg TE/g and 32.51 ± 0.64 mg TE/g, respectively). Likewise, it's worth noting that the DPPH values for leaves were higher than those for stem and roots. The variation in DPPH values among different parts of *S. cumini* suggests that each part may contribute differently to the overall antioxidant potential of the plant. The DPPH values reported in this study for *S. cumini* are comparable to findings in other plant studies. For instance, previous research on papaya peel resulted in a strong DPPH scavenging rate of 94.79% ± 0.10% (Pei et al. 2022). The presence of carotenoids, lycopene, sterols, unsaturated fatty acids, and phenols in plant peels has been linked to their higher free radical scavenging capacity (Ugbabe et al. 2010). The ABTS assay, a commonly employed method to estimate the antioxidant ability of phenolic compounds, was utilized to assess the antioxidant potential of different parts of *S. cumini* . Table 1 provides the ABTS values for the stem, roots, and leaves. The results revealed that the leaves of *S. cumini* performed the highest ABTS value (24.59 ± 0.899 mg AAE/g), implying a substantial hydrogen atom donation tendency and potent antiradical scavenging activity. In comparison, the stem and roots displayed lower ABTS values (22.88 ± 0.911 mg AAE/g and 20.57 ± 0.199 mg AAE/g, respectively). ABTS values demonstrated variations among the different parts of *S. cumini* , emphasizing the differential contribution of each part to the overall antioxidant capacity. Notably, leaves may predominate with their higher ABTS values in enhancing the plant's antioxidant potential. The ABTS assay results align with the general trend observed in the DPPH assay, where leaves consistently exhibited higher antioxidant values compared to the stem and roots. This suggests that the leaves of *S. cumini* may contain a richer concentration of phenolic compounds.

The rest of the antioxidant assays, including FRAP, RPA, ·OH‐RSA, FICA, and TAC assays, were also used to assess a comprehensive evaluation of the antioxidant potential of *S. cumini* . The specific values for these assays are provided in Table 1. The FRAP values revealed that the different parts of *S. cumini* exhibited varying ferric‐reducing abilities. For instance, the leaves showed the highest FRAP value (59.61 ± 0.941 mg TE/g), followed by the stem and roots. This indicates the capacity of phenolic compounds in the leaves to reduce ferric ions, contributing to the overall antioxidant mechanism.

The RPA values also demonstrated variations among the plant parts, with the leaves exhibiting the highest reducing power (71.23 ± 6.92 mg TE/g). The stem and roots displayed lower RPA values, suggesting differences in their potential to reduce ferric ions. The ·OH‐RSA values, reflecting hydroxyl radical scavenging activity, were not reported for leaves, indicating that this specific assay was not conducted for this plant part. However, the stem and roots displayed ·OH‐RSA values of 87.57 ± 7.51 mg AAE/g and 348.52 ± 18.55 mg AAE/g, respectively, suggesting their capability to scavenge hydroxyl radicals. Similarly, the FICA values indicated variations among the plant parts, with the leaves exhibiting the highest FICA value (87.57 ± 7.51 mg EDTA/g). The stem and roots displayed lower FICA values, suggesting differences in their ability to reduce ferric ions. The TAC values, representing the overall antioxidant capacity, demonstrated that the different parts of *S. cumini* exhibited varying levels of total antioxidants. The leaves showed the highest TAC value (5.99 ± 0.001 mg TE/g), reflecting their significant contribution to the overall antioxidant potential of the plant.

*S. cumini* , commonly known as Jamun, has extensively explored its antioxidant potential using diverse assays, including but not least DPPH, FRAP, and ABTS assays. Earlier, Banerjee & Dasgupta (Banerjee et al. 2005) carried out a detailed investigation of the antioxidant potential of *S. cumini* fruit through different assays. The study found that the *S. cumini* fruit skin‐based extract showed the strongest antioxidant activity, with an IC_50_ value of 428 μg/mL. From their perspective, the *S. cumini* fruit skin contains water‐soluble scavengers that effectively counteract the highly toxic superoxide radicals (O^2−^) generated in various biological and photochemical reactions. Likewise, Ruan et al. (Ruan et al. 2008) explored the antioxidant potential of *S. cumini* leaf extracts, which showed significant activity. Likewise, Benherlal & Arumughan (Benherlal and Arumughan 2007) stated that the different fractions of *S. cumini* like pulp, kernel, and seed coat showed promising radical scavenging and antioxidant capacity in an order of effectiveness as seed > kernel > pulp.

*S. cumini's* remarkable antioxidant activity may account for its rich composition of bioactive compounds, particularly polyphenols such as anthocyanins, ellagic acid, and tannins. These antioxidants act as potent scavengers of free radicals, including the superoxide radical (O^2−^), generated during biological and photochemical reactions (Singh et al. 2016). Anthocyanins, responsible for the fruit's vibrant color, are well known for their free radical‐quenching properties, while ellagic acid and tannins contribute additional antioxidant effects. The synergistic action of these bioactive compounds makes *S. cumini* a promising natural source for combating oxidative damage and suggests its potential application in preventive and therapeutic strategies against oxidative stress‐related disorders (Pino et al. 2022).

### 3.2 Correlating the Antioxidant Potential With Polyphenols

The Pearson's correlation coefficient (*r*) in Table 2 reveals significant associations between various assays measuring antioxidant capacity and phenolic contents in different parts of *S. cumini* . Notably, TFC exhibits a strong positive correlation (0.50 < *r* < 0.80, *p* < 0.01) with TPC and TTC, emphasizing the interconnectedness of flavonoid and phenolic compounds. The DPPH assay shows a moderate positive correlation with TPC, TFC, and TTC, suggesting a shared variability in their antioxidant potential. Furthermore, the FRAP and ABTS assays display robust positive correlations with TPC, TFC, and TTC, underscoring their close relationship with phenolic contents. ·OH‐RSA exhibits positive correlations with various parameters, including TPC, TFC, TTC, DPPH, FRAP, and ABTS, indicating its broad association with antioxidant assays and phenolic compounds. In contrast, the RPA demonstrates a highly significant negative correlation with TPC, TFC, TTC, DPPH, FRAP, and ABTS, suggesting an inverse relationship with the measured antioxidant assays. These correlation findings provide valuable insights into the interplay between different antioxidant assays and phenolic compounds in *S. cumini* .

**TABLE 2:** Pearson's correlation coefficient (*r*) for the relationships between assays for antioxidant capacity and phenolic content.

Variables	TPC	TFC	TTC	DPPH	FRAP	ABTS	·OH‐RSA	RPA	FICA
TFC	0.796								
TTC	0.828*	0.999**							
DPPH	0.935*	0.959*	0.973*						
FRAP	0.330*	0.309*	0.257*	0.025*					
ABTS	0.842*	0.997*	1.000**	0.978*	0.232*				
·OH‐RSA	0.963*	0.605*	0.646*	0.806*	0.571	0.666			
RPA	−0.399*	−0.872*	−0.845*	−0.697*	0.734*	−0.831	−0.138		
FICA	−0.773	−0.999	−0.996	−0.947	0.345	−0.993	−0.574	0.891	
TAC	0.986	0.886	0.909	0.981	0.168	0.920	0.905	−0.546	−0.867

*Note:* *Represents significant correlation at *p* < 0.05; **Represents a highly significant correlation at *p* < 0.01.

**FIGURE 1:**
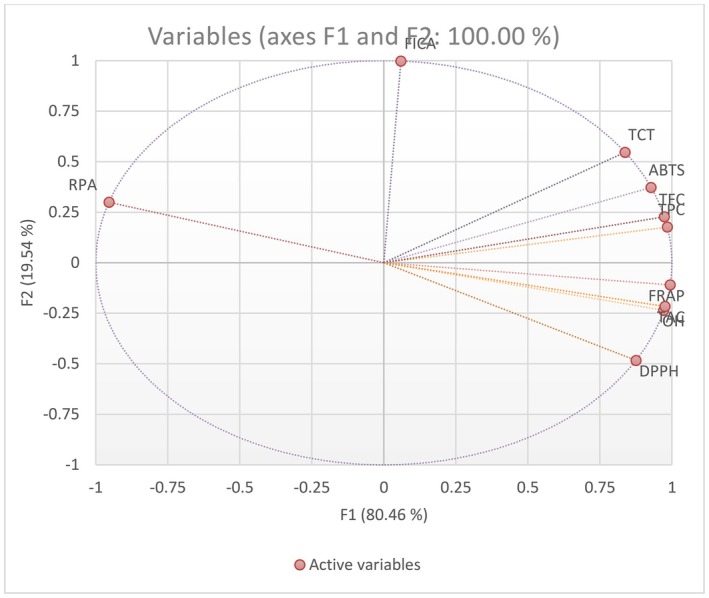
Principal component analysis (PCA) graph shows the correlation between the samples.

In addition, the inter‐relationships among the three characterizations and seven antioxidant assays were explored using PCA, as shown in Figure 1. The first principal factor (F1) accounted for 80.46% of the variability, while the second factor (F2) explained 19.54% of the variability. Most assay results were observed to exhibit a positive trend along the x‐axis, including three characterization assays (TPC, TFC, and TTC). TPC is highly correlated with DPPH, TAC, and ·OH‐RSA; nevertheless, TFC and TTC are significantly correlated (*p* < 0.05) with ABTS.

### 3.3 Characterization

The LC‐ESI‐QTOF‐MS/MS analysis of *S. cumini* extracts yielded a comprehensive characterization of phenolic compounds in stem, root, and leaf samples, and 12 compounds were identified on different ionization modes and *m/z* ratios; all these data were performed in Table 3.

**TABLE 3:** Characterization of phenolic compounds detected in conventional and ultrasound extracts of stem, root, and leaf of *S. cumini* by LC‐ESI‐QTOF‐MS/MS.

No.	Proposed compounds	Molecular Formula	RT (min)	Ionization (ESI^+^/ESI^−^)	Molecular Weight	Theoretical (*m/z*)	Observed (*m/z*)	Error (ppm)	MS^2^ Product ions	*Syzygium cumini* samples
Phenolic acid										
Hydroxybenzoic acids										
1	3,4‐*O*‐Dimethylgallic acid	C_9_H_10_O_5_	18.322	[M + H]+	198.0534	199.0607	199.0605	−1.0	153, 139, 125, 111	SCS
2	Protocatechuic acid	C_7_H_6_O_4_	18.129	[M‐H]−	154.0255	153.0182	153.0181	−0.7	153	SCS
Hydroxycinnamic acids										
3	Hydroxycaffeic acid	C_9_H_8_O_5_	4.922	[M‐H]−^b^	196.0387	195.0314	195.0313	−0.5	151	SCS
Flavonoids										
Flavanols										
4	Scutellarein	C_15_H_10_O_6_	26.39	[M + H]+	286.0482	287.0555	287.056	1.7		SCS, SCR, SCL^a^
Anthocyanins										
5	Pelargonidin 3‐*O*‐rutinoside	C_27_H_31_O_14_	24.286	[M‐H]−	480.0951	479.0878	479.0833	−9.4	433,271	SCS
Isoflavonoids										
6	5,6,7,3′,4’‐Pentahydroxyisoflavone	C_15_H_10_O_7_	25.126	[M + H]+	302.0446	303.0519	303.0529	3.3	285, 257	SCS
Flavanones										
7	Naringin 4’‐*O*‐glucoside	C_33_H_42_O_19_	34.39	[M‐H]−	742.2353	741.2280	741.2276	−0.5	271	SCS
Other polyphenols										
Hydroxycoumarins										
8	Scopoletin	C_10_H_8_O_4_	6.972	[M‐H]−	192.0421	191.0348	191.0349	0.5	171	SCS, SCL^a^
Tyrosols										
9	3,4‐DHPEA‐EDA	C_17_H_20_O_6_	4.589	[M‐H]−^b^	196.0742	195.0669	195.067	0.5	275, 195	SCS, SCR, SCL^a^
10	Demethyloleuropein	C_24_H_30_O_13_	5.171	[M + H]+	526.171	527.1783	527.1792	1.7	495	SCS, SCR, SCL^a^
Lignans										
11	Schisandrin C	C_22_H_24_O_6_	23.182	[M + H]+	384.1542	385.1615	385.1611	−1.0	370, 315, 300	SCR
12	Schisandrin B	C_23_H_28_O_6_	25.558	[M + H]+	400.1891	401.1964	401.1965	0.2	224, 193, 165	SCS

^a^ Compound was detected in more than one sample, data presented in this table are from an asterisk sample.

^b^ Compounds were detected in both negative [M‐H]^−^ and positive [M + H]^+^ modes of ionization while only single mode data was presented.

Abbrevciations: SCL, *Syzygium cumini* Leaf; SCR, *Syzygium cumini* root; SCS, *Syzygium cumini* Stem.

### 3.4 Phenolic Acid

In the analysis of *S. cumini* , three phenolic compounds were identified and classified into two classes: hydroxybenzoic acids and hydroxycinnamic acids. Among the hydroxybenzoic acids, Compound 1, identified as 3,4‐*O*‐Dimethylgallic Acid (*m/z* 199.0605), was detected in conventional extracts of stem, root, and leaf samples. This compound was detected at positive mode with the observed m/z at 199.0605, which is consistent with the finding of Shi et al. (Shi et al. 2024). Compound 2, protocatechuic acid (*m/z* 153.0181), was found in conventional extracts of stem and root samples. In the hydroxycinnamic acids category, Compound 3, hydroxycaffeic acid (*m/z* 195.0313), was identified in stem samples. The results highlight the presence of specific phenolic compounds in different plant parts, exhibiting precious insights into the phenolic composition of *S. cumini* .

Previous studies on medicinal plants have identified hydroxybenzoic and hydroxycinnamic acids as common phenolic constituents (Zhou et al. 2024). Like our findings, the presence of compounds such as protocatechuic acid and hydroxycaffeic acid has been reported in several plant species, contributing to antioxidant and anti‐inflammatory properties (Wei et al. 2024). Protocatechuic acid performed a cardioprotective function (Yeniçeri et al. 2024), as well as anti‐inflammatory, antihyperglycemic, and antiapoptotic activities by in vivo experiments using rats and mice, as well as inhibiting chemical carcinogenesis (Semaming et al. 2015). Furthermore, the distribution of phenolic compounds across different plant parts, as observed in our study, aligns with findings from investigations into the phytochemical composition of various botanical sources. The variations in phenolic content among stems, roots, and leaves have been attributed to differences in the plant's metabolic processes and environmental factors. Additionally, the use of LC‐ESI‐QTOF‐MS/MS for phenolic compound analysis, as employed in our study, has been recognized for its accuracy and sensitivity in identifying and quantifying plant metabolites. Comparable analytical techniques have been applied in previous studies, enhancing the reliability and comparability of our results with existing literature (Chu et al. 2023).

3,4‐*O*‐Dimethylgallic acid, also known as gallic acid dimethyl ether, is a derivative of gallic acid. Gallic acid is a trihydroxybenzoic acid with three hydroxyl (OH) groups located at positions 3, 4, and 5 on the benzene ring. The synthesis of 3,4‐*O*‐dimethylgallic acid involves the methylation of the hydroxyl groups at positions 3 and 4 of the gallic acid molecule. This chemical modification introduces two methyl (CH_3_) groups to the gallic acid structure. The formation of 3,4‐*O*‐dimethylgallic acid typically involves a methylation reaction, where methyl groups are added to specific hydroxyl groups of the gallic acid molecule, whereas protocatechuic acid is a dihydroxybenzoic acid with two hydroxyl (OH) groups positioned at positions 3 and 4 on the benzene ring. It is structurally related to catechol and is a natural phenolic compound (Duan et al. 2023). The results regarding the identification of 3,4‐*O*‐dimethylgallic acid and protocatechuic acid are in line with the earlier findings of Lyu et al. (Lyu et al. 2023) observed the 3,4‐*O*‐dimethylgallic acid and protocatechuic acid in a similar ionization mode (** [M + H]^+^ &) and *m/z* ratios, 199.0598 & 315.0718, respectively.

Both 3,4‐*O*‐dimethylgallic acid and protocatechuic acid offer notable health benefits. 3,4‐*O*‐dimethylgallic acid, derived from the methylation of gallic acid, exhibits significant antioxidant properties. This suggests its potential in combating oxidative stress and inflammation, which are implicated in various chronic health conditions. On the other hand, protocatechuic acid, a dihydroxybenzoic acid found in fruits and vegetables, pertains to health‐promoting effects. It demonstrates anti‐inflammatory properties and has been explored for its potential in managing diabetes (Kumar et al. 2022).

### 3.5 Flavonoids

In the analysis of *S. cumini* , a total of four flavonoid subgroups were identified, each containing specific compounds. Within the flavanols subgroup, scutellarein (*m/z* 287.056) was detected in all samples under positive mode. Scutellarein has therapeutic effects on tumors, Alzheimer's disease, and obesity and inhibits the NF‐κB signaling pathway to treat the β‐amyloid lesion (Huang et al. 2019; Liu et al. 2023; Miao et al. 2020).

Moving to the anthocyanin subgroup, pelargonidin 3‐*O*‐rutinoside (*m/z* 479.0833) was identified in the root sample (SCR). The pelargonidin 3‐*O*‐rutinoside from strawberry is a novel α‐glucosidase inhibitor (Xu et al. 2019). In the isoflavonoids subgroup, 5,6,7,3′,4′‐pentahydroxyisoflavone (*m/z* 303.0529) was identified in the stem sample (SCS), highlighting the presence of compounds with potential estrogenic activity. Lastly, in the flavanone subgroup, Naringin 4'‐O‐glucoside (*m/z* 741.2276) was detected in the stem sample (SCS) in the negative mode, and this is in line with previous research (Liu et al. 2024).

Flavonoids, a diverse group of polyphenolic compounds abundantly and widely found in fruits, vegetables, tea, and various plant‐based foods, have gained substantial attention because of their multifaceted functions and potential health benefits (Zhou et al. 2023a, 2023b). These compounds are recognized for their antioxidant properties, involving neutralizing free radicals and reducing oxidative stress within the body. Beyond their antioxidant activity, flavonoids are known to possess anti‐inflammatory, anti‐cancer, and cardiovascular protective effects. Quercetin, found in apples and onions, for instance, exhibits anti‐inflammatory properties, while catechins in green tea have been associated with cardiovascular health. The diverse functions of flavonoids extend to their potential to enhance cognitive function and decrease the rate at which neurodegenerative diseases occur (Wang et al. 2023).

The compound scutellarein is a flavone derived from the flavonoid scutellarin and features a distinctive chromone ring structure with hydroxyl groups, showcasing its unique chemical composition. Typically formed through the hydrolysis of scutellarin and known for its antioxidant properties, scutellarein is recognized for its potential to neutralize free radicals and protect against oxidative stress (Spiegel et al. 2022).

Naringin 4'‐O‐glucoside, a flavonoid glycoside derived from citrus fruits, consists of a flavonoid aglycone linked to a glucose molecule at the 4' position. Its formation involves enzymatic glycosylation processes within plants. It is recognized for its antioxidant and anti‐inflammatory properties (Sukmaningsih et al. 2019). According to previous studies, substance N exhibits anti‐cancer functions such as inhibiting cell proliferation and promoting cell apoptosis (Stabrauskiene et al. 2022).

The identification of compounds in the current study as different classes of flavonoids is in harmony with earlier findings using LC‐ESI‐QTOF‐MS/MS for the identification of different phenolics, and similar patterns were observed in different fruits and their fractions. They stated that the concentration varied among the other parts. However, the compound's presence depends upon the extraction module, solvent, and type of fraction (Lyu et al. 2023; Shi et al. 2023). Earlier, (Priya et al. 2017) found phenolic acids and flavonoids in three different varieties of *S. cumini* seeds and observed that gallic acid, ferulic acid, and caffeic acids were the most abundant ones, validating the strong antioxidant profile of *S. cumini*.

### 3.6 Other Polyphenols

In the category of “Other Polyphenols” within *S. cumini* , a total of three compounds were identified, each belonging to distinct subclasses. These subclasses include hydroxycoumarins and tyrosols. Scopoletin, belonging to hydroxycoumarins, exists in the stem and leaf sample. The observed *m/z* is 191.0349 [M‐H]^−^. The results are consistent with the previous study (Zhou et al. 2023a, 2023b). 3,4‐DHPEA‐EDA, compound 9, was detected in both negative and positive modes with the observed 195.067 *m/z*. Lastly, compound 10, demethyloleuropein, a tyrosol, showed a *m/z* of 527.1792 [M + H]^+^ and was identified in all samples.

Scopoletin is formed by substituting the lactone with a hydroxyl group (OH) at the 7th position on the benzopyran ring. This hydroxyl group is instrumental in shaping its biological activities and potential pharmacological properties. Extensive research has explored the diverse effects of scopoletin, encompassing antioxidant and anti‐inflammatory activities (Tiwari et al. 2023). 3,4‐DHPEA‐EDA is an aglycone derived from oleuropein; it is also a major polyphenol derived from olive leaves. According to the research of Takashi et al. (Akazawa et al. 2021), the texture of the protein gel would be improved by the 3,4‐DHPEA‐EDA extracted through cold water.

### 3.7 Lignans

In the category of “Lignans” within *S. cumini* , two compounds were identified, each belonging to distinct subclasses. These subclasses include Schisandrin C (Compound 11) and Schisandrin B (Compound 12).

Schisandrin C, a lignan with a *m/z* of 385.1611 [M + H]^+^, was consistently present in the root samples (SCR). The detection of schisandrin C in the analyzed samples underscores its prevalence in the stem fraction of *S. cumini* . Schisandrin B, another lignan with a *m/z* of 401.1965 [M + H]^+^, was identified in the stem samples (SCS). The presence of Schisandrin B in the stem fraction further contributes to the characterization of lignans in *S. cumini* .

Schisandrin C, a lignan compound sourced from *Schisandra chinensis* fruit, is formed through specific biosynthetic pathways within the plant. Renowned for its adaptogenic qualities, it enhances stress resistance and overall well‐being. This compound's antioxidant properties shield cells from oxidative damage, and studies suggest hepatoprotective effects, supporting liver health. Its anti‐inflammatory attributes further indicate potential applications in addressing inflammatory conditions (Zaki et al. 2023).

Identifying these lignans in distinct plant parts provides insights into the phytochemical composition of *S. cumini* . Lignans caught sustainable attention because of their potential health benefits, such as antioxidant and anti‐inflammatory properties, and their presence in this plant species suggests their potential contribution to its medicinal properties. Further studies on the specific roles and bioactivities of these lignans can enhance our understanding of the therapeutic potential of *S. cumini* .

In conclusion, our findings on phenolic compounds in *S. cumini* align with broader trends observed in plant research. The presence of specific compounds and their distribution among different plant parts is consistent with the existing knowledge of phytochemical diversity.

### 3.8 Distribution of Phenolic Compounds

**FIGURE 2:**
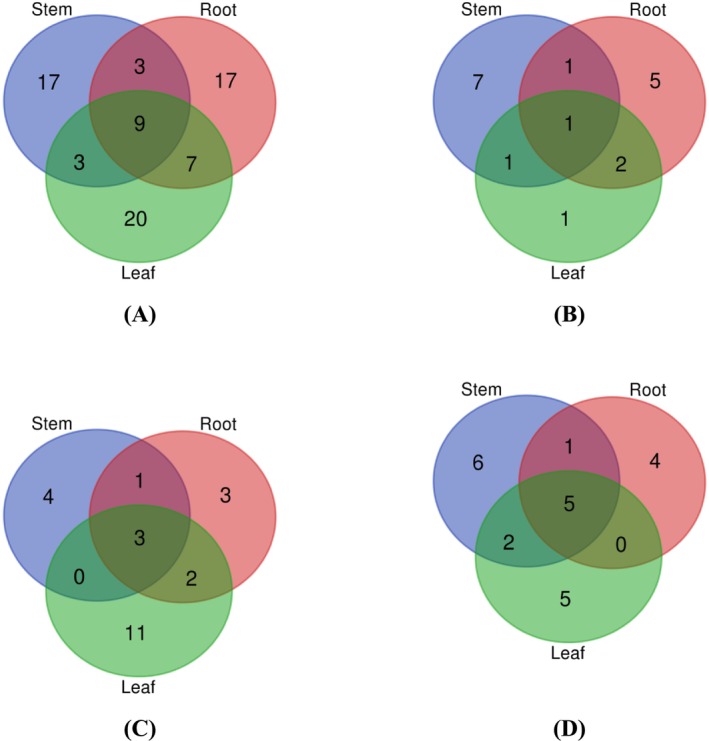
Venn diagram of screened phenolic compounds species present in different parts (Stem, root, and leaf) stem, root, and leaf of *S. cumini* . (A) distribution of all the screened phenolic compounds in different parts (Stem, root and leaf) of *S. cumini* (B) distribution of phenolic acids in different parts (Stem, root and leaf) of *S. cumini* (C) focuses on the lignans and stilbenes across the different plant parts (D) distribution of other polyphenols (including lignans and stilbenes) different parts (Stem, root and leaf) of *S. cumini* .

Venn diagrams were utilized to assess the distribution of phenolic compounds within distinct parts (stem, root, and leaf) of *S. cumini* , offering a visual representation of compound overlap and uniqueness based on the identification results through LC‐ESI‐QTOF‐MS/MS. In Figure 2, Panel (A) illustrates the overall distribution of all screened phenolic compounds across the various plant parts, revealing that 28.95% of the total 76 compounds of the identified phenolic compounds were common across all parts. Panel (B) of the Venn diagram highlights the distribution of phenolic acids within the stem, root, and leaf, indicating that 27.78% (5 compounds) were shared among these parts; however, the stem exhibited the highest number (7) of different phenolic acids compared to the root (5) and leaf (1). Similarly, Panel (C) focuses on the lignans and stilbenes across the different plant parts, revealing that a total of 24 compounds were identified, with 6 found common, and the leaf exhibited the highest individual compounds (11). This visual analysis provides insights into both the shared and unique phenolic compound species in the studied plant components, contributing to a nuanced understanding of *S. cumini's* phytochemical composition.

Moreover, Venn diagrams explore the distribution of phenolic compounds in different ripeness stages, color variations, and fruit parts. In this context, they provide a comprehensive breakdown of compound distribution, allowing for a detailed examination of commonalities and disparities among different plant samples. The incorporation of percentages and numbers enhances the clarity of the findings, providing a quantitative perspective on the prevalence of phenolic compounds in *S. cumini* .

## 4 Conclusions

In summary, this study presents promising insights regarding the antioxidant and phenolic profile of the *S. cumini* leaf, root, and stem. In this study, we might be the first to utilize the ten different assays to evaluate the polyphenol compounds along with their antioxidant potential of this plant. Leaves performed better in phenolics and antioxidant activity among the different fractions, followed by stem and root. The LC‐ESI‐QTOF‐MS/MS identification showed the presence of 12 phenolic compounds belonging to the different classes of polyphenols. However, future research focusing on bioaccessibility, bioavailability, toxicology, and animal models is recommended to enhance the possibilities of leaf, stem, and root‐based commercial products in the market.

## Author Contributions

**Ali Imran:** conceptualization (equal). **Shujun Ye:** formal analysis (equal). **Jiaying Amanda Li:** formal analysis (equal). **Rahaf Ajaj:** provided the methodology. **Abdur Rauf:** writing – original draft (equal), writing – review and editing (equal). **Zubair Ahmad:** software (equal), writing – review and editing (equal). **Hassan A. Hemeg:** formal analysis (equal). **Yahya Saleh Mohamed Al‐Awthan:** formal analysis (equal). **Omar S. Bahattab:** formal analysis (equal). **Mohammed Mansour Quradha:** supervision (equal). **Hafiz Suleria:** formal analysis (equal), validation (equal), writing – original draft (equal), writing – review and editing (equal).

## Conflicts of Interest

The authors declare no conflicts of interest.

## Data Availability

The spectroscopic data and other physical data of compounds associated with this manuscript are available from the corresponding author upon request.
